# Social and non-social categorisation in investment decisions and learning

**DOI:** 10.1177/17470218231153137

**Published:** 2023-02-04

**Authors:** Maïka Telga, José A Alcalá, Juan Lupiáñez

**Affiliations:** 1 Department of Experimental Psychology, University of Granada, Granada, Spain; 2 School of Management, University of St Andrews, St Andrews, UK; 3 University of Jaén, Jaén, Spain; 4 Complutense University of Madrid, Madrid, Spain

**Keywords:** Trust game, learning, individuation, categorisation, economic reward

## Abstract

Categorical processes allow us to make sense of the environment effortlessly by grouping stimuli sharing relevant features. Although these processes occur in both social and non-social contexts, motivational, affective, and epistemic factors specific to the social world may motivate individuation over categorisation of social compared with non-social stimuli. In one experiment, we tested this hypothesis by analysing the reliance on categorical versus individuating information when making investment decisions about social and non-social targets. In an adaptation of the iterative trust game, participants from three experimental groups had to predict the economic outcomes associated with either humans (i.e., social stimuli), artificial races (i.e., social-like stimuli), or artworks (i.e., non-social stimuli) to earn economic rewards. We observed that investment decisions with humans were initially biased by categorical information in the form of gender stereotypes, but later improved through an individuating learning approach. In contrast, decisions made with non-social stimuli were initially unbiased by categorical information, but the category–outcomes associations learned through repeated interactions were quickly used to categorise new targets. These results are discussed along with motivational and perceptual mechanisms involved in investment decisions and learning about social and non-social agents.

Successful learning about people and objects depends on our capacity to exploit the contingencies of our environment both in social and non-social contexts. For instance, discriminating between trustworthy and untrustworthy interactions partners is critical for harmonious relationships. In the financial domain, being able to accurately assess the value of a particular company over time is essential for successful investments. In both scenarios, through repeated interactions, we learn by trial and error what stimuli to approach and which ones to avoid, a process that has been conceptualised as reinforcement learning (Sutton & Barto, 1981, 2018).

Economics games such as the Trust Game (Berg et al., 1995) have been revisited to recreate in the lab cooperation dynamics (King-Casas et al., 2005) that resemble reinforcement learning. In an iterated trust game, earning monetary rewards requires observing and reacting to the actions of unfamiliar game partners across trials (Alós-Ferrer & Farolfi, 2019). Although in such games, we typically try to exploit as much as possible the information available to us to establish relationships between stimuli and outcomes, our decisions are often biased by cognitive and social biases (Dunning et al., 2012; van den Berg et al., 2021; Vermue et al., 2018). In the present research, we investigate whether a categorisation bias differentially impacts learning about social versus non-social stimuli.

## Categorisation and individuation processes

Categorisation consists of grouping together stimuli that share relevant and salient features, allowing perceivers to bring coherence to the diversity of stimuli surrounding them, including people (Simon, 1993), objects (Murphy, 2010), artworks (dos Santos Ferreira et al., 2018), or companies (Krüger et al., 2012). For instance, perceivers may categorise seats with four legs and a back as chairs and use them adequately without attending to the differences between and uniqueness of all exemplars of chairs. However, categorical processes sometimes lead to flawed decisions and economic loss (e.g., Telga et al., 2018). If an entire sub-group of elements (e.g., artworks by Artist A) is perceived of a higher value than a second sub-group (e.g., artworks by Artist B), people may base their financial decisions on their knowledge or beliefs related to these categories (e.g., investing more in artworks from Artist A than Artist B), instead of attending the individual value of each particular painting. Given that the value of a specific painting from Artist B could overpass the value of a specific painting from Artist A, this decision will sometimes be inaccurate (Mullainathan, 2002).

Categorisation is also a key process in social perception. On the basis of easily noticeable features related to their group membership (e.g., skin tone), people often spontaneously categorise others to make complex inferences about them (Blair et al., 2002; Bodenhausen et al., 2012; Fiske & Neuberg, 1990; Macrae & Bodenhausen, 2000). For instance, if a perceiver believes that women are more trustworthy than men (Buchan et al., 2008; Cuddy et al., 2008), they may make assumptions about a particular person’s trustworthiness on the basis of the facial features informative of their sex (Telga et al., 2023).

Nevertheless, under some circumstances, perceivers may further their level of analysis beyond category-related information by attending individual attributes informative of a person’s identity (Neuberg & Fiske, 1987; Rodriguez-Bailon et al., 2006). For instance, they may monitor a target’s behaviours and decide whether or not this person may be trusted independently of their gender. This approach comes at a greater cognitive cost (Gilbert & Hixon, 1991; Macrae et al., 1994), but also offers more accurate judgements. Depending on the context, perceivers may therefore be more prone to either individuate or categorise stimuli.

## Social versus non-social stimuli

In economic decision-making, one of the factors that may impact responses is whether they involve social or non-social stimuli. Comparing financial decisions with humans versus non-human agents, researchers have highlighted different patterns of responses at the behavioural (see March, 2021 for a review), affective (Schniter et al., 2020), and neural (Hackel et al., 2015) levels. Here, we adopt a similar approach and explore the impact of categorisation and individuation processes on financial decisions with social and non-social stimuli. We argue that although social and non-social categorisations respond to the same need for cognitive efficiency, they also differ in several aspects that may promote a more individuating approach for social compared with non-social stimuli.

First, once social categories are established, perceivers immediately identify the groups they fall into (i.e., ingroups) and the groups they do not belong to (i.e., outgroups). These self-categorisation processes are associated with profound psychological changes at the motivational, cognitive, and affective levels (Ellemers & Haslam, 2012; Tajfel & Turner, 1979; Turner et al., 1987). For example, ingroups are perceived as more heterogeneous than outgroups, and ingroup members are typically approached in an individuated manner (Telga et al., 2018), an effect that has been conceptualised as outgroup homogeneity (Haslam et al., 1996; Simon, 1993). The individuating processes resulting from social identification are, by definition, reserved for social stimuli.

Second, from the first years of life, humans are able to adopt others’ perspectives and empathise with them (Frith & Frith, 1999; Shamay-Tsoory, 2011). These affective processes also impact social perception by decreasing attention to category-related and stereotypic features (Galinsky & Moskowitz, 2000) in favour of a more individuated perception of the target. However, such personal involvement seems far less likely with non-social stimuli (Schniter et al., 2020).

Finally, epistemic motivation influences social perception and may explain inter-individual differences regarding how this social knowledge is acquired (Bodenhausen et al., 2006; Chuah et al., 2014; Ford & Kruglanski, 1995), in particular in economic contexts (Fehr & Schmidt, 1999; Tasch & Houser, 2018). In the non-social realm, however, people may be less concerned with epistemology and adopt a more reward-oriented approach. For example, Hackel and colleagues found that when presented with the same stimuli–outcomes contingencies, participants relied more on monetary rewards (i.e., quantity shared independently of the amount possessed) when they believed they played with machine slots, but more on feedback indicating trait generosity (i.e., the willingness to share a large amount of what is possessed) when they believed they played with humans (Hackel et al., 2015, 2020)

In sum, self-perception, affective factors, and epistemic concerns converge to predict an inclination to understand people, but not objects, as unique individuals; a prediction tested in the present research.

## The present research

Building on the revised literature, we explored whether financial decisions with human targets are more likely based on individuating information than financial decisions with non-social stimuli. In addition, we introduced a “social-like” condition to explore where do stimuli that share physical characteristics with humans (i.e., similar facial features) but are clearly not human, fall on the categorisation–individuation continuum. For this, we used an adaptation of the trust game in which participants were assigned to one out of three experimental groups: social (i.e., humans), social-like (i.e., artificial races), or non-social (i.e., artworks) stimuli. Artworks were chosen in the non-social group because they are perceptually complex and categorisable (dos Santos Ferreira et al., 2018). Each phase of the Trust Game was designed to explore the mechanisms underlying investment decisions in three different contexts: (1) the baseline phase examined spontaneous investments with unfamiliar targets, (2) the learning phase investigated learning of specific (categorical and/or individual) associations between targets and outcomes across repeated interactions, and (3) the transfer phase assessed whether the learned information acquired during the learning phase would be transferred to new targets in a categorical fashion. Because categorisation and individuation may differentially impact decisions in these three contexts, we formulated specific hypotheses for each phase of the trust game.

In the baseline phase, we expected participants playing with humans to be driven by gender stereotypes and to cooperate more with female than with male partners (Brañas-Garza et al., 2018; Buchan et al., 2008) (Hypothesis 1a). With social-like and non-social stimuli, however, we did not expect any prior knowledge to bias participants’ responses and, therefore, expected similar investment rates with the two categories of social-like (Hypothesis 1b) and the two categories of non-social (Hypothesis 1c) stimuli.

In the subsequent learning phase, we expected participants to individuate social targets (Hypothesis 2a) and to categorise both social-like (Hypothesis 2b) and non-social (Hypothesis 2c) stimuli.

Finally, in the final transfer phase, we expected some expression of categorical learning in all conditions (Hackel et al., 2021; Vermue et al., 2019). In particular, we predicted that participants would generalise the category–reward associations learned in the learning phase to new targets with whom they have no prior experience. Hence, we expected all participants to invest more in the category that was associated with positive outcomes in the learning phase when playing with new social (Hypothesis 3a), social-like (Hypothesis 3b) and non-social (Hypothesis 3c) stimuli.

## Method

### Participants

One hundred twenty participants (18 men, mean age: 22.28, range: 18–45) took part in the study in exchange for a financial compensation ranging from €0 to €10, proportional to their accuracy in the task (€5.85 on average). Sample size was based on Telga et al. (2018), as this sample proved sensitive to detect differences in categorising vs. individuating approach in spontaneous investments and learning in the Trust Game. Furthermore, sensitivity analyses revealed that with the sample of 120 participants, the smallest effect size that could be detected in the learning phase for the critical Dimension × Consistency × Block interaction with a power of .80 and an alpha criterion of .05 was $\eta_{\text{p}}^{2}$ = .04. Note that the observed effect ( $\eta_{\text{p}}^{2}$ = .05, see below) is larger than .04. For this effect, the actual observed power was .88. This study is part of a larger research project approved by the local university research ethics committee (175/CEIH/2017).

### Stimuli and materials

For the social condition, the stimuli were human targets belonging to one of two gender groups: men or women. For the social-like condition, we used pictures of individuals sharing facial features with humans, and could be categorised into one of two artificial races: Lunaris and Taiyos. Finally, for the non-social condition, the stimuli were artworks from two different artists with unequivocally different styles: Wassily Kandinsky and Jaison Cianelli. In the present work, social (i.e., humans), social-like (i.e., artificial races), and non-social (i.e., artworks) conditions are referred to as “dimensions.” Within these dimensions, men vs. women, Lunaris vs. Taiyos, and Kandinsky vs. Cianelli are referred to as “categories,” and exemplars within these categories are referred to as “individuals” or “targets.”

Several pilot experiments were conducted with different sets of stimuli to select the targets to be used in the trust game ensuring that the stimuli were categorisable into two categories. Details on stimuli selection are available in Section 3 of the Online Supplementary Material. We selected 32 photographs of men and women from the Karolinska Direct Emotional Faces (Lundqvist et al., 1998) for the group playing with the social dimension (i.e., humans), 32 pictures of Lunaris and Taiyos (Chua et al., 2014) ceded by the Object Perception Lab of Vanderbilt University for the group playing with social-like targets (i.e., artificial races), and 32 images of artworks from Wassily Kandinsky and Jaison Cianelli for the group playing with the non-social stimuli.

### Procedure

**Figure 1.**
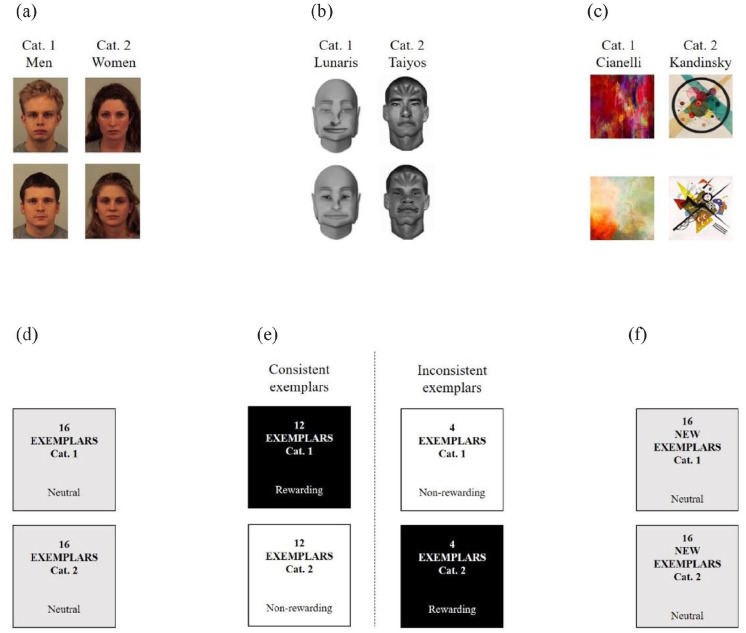
Example of stimuli (a) used in the groups playing with humans, (b) artificial races, and (c) artworks, and procedure employed in the (d) baseline, (e) learning, and (f) transfer phases. Exemplars are represented in black when they are rewarding (i.e., they allow participants to earn economic outcomes on 75% of the trials), in white when they are non-rewarding (i.e., they make participants lose money on 75% of the trials), and in grey when they are associated to positive economic outcomes on half of the trials, and to economic loss on the other half.

Upon arrival to the laboratory, participants provided written consent and were led to individual cubicles. All participants first received brief verbal instructions (see Section 1.1. of the Online Supplementary Material) about the general structure of the experiment and their financial compensation (i.e., how it was calculated and what was the maximum they could earn). They were also explained that the scenarios depicted in the experiments were fictitious but were asked to try to immerse themselves in these scenarios as best as possible. Participants were randomly assigned to play with either humans (16 men and 16 women), artificial races (16 Lunaris and 16 Taiyos), or artworks (16 Cianelli’s and 16 Kandinsky’s) in a modified iterated trust game (Telga et al., 2018) (see Figure 1). At this point, they received specific visual instructions depending on the experimental condition they were in (see Section 1.2. of the Online Supplementary Material). Specifically, participants playing with humans and artificial races were instructed to decide whether or not to share their money with several game partners (see Section 1.2.1 of the Online Supplementary Material), while participants playing with artworks were instructed to decide whether or not to invest their money in different artworks (see Section 1.2.2 of the Online Supplementary Material).

Each trial started with the euro symbol “€” (1.43º × 1.63º) for 190 ms representing that participants virtually received €1, followed by a fixation point in the centre of the screen for 500 ms. Next, the picture of the target of this trial appeared for 1,500 ms (5.68º × 7.77º) and participants had to decide whether or not to invest €1 in this trial by pressing “1” to invest or “0” not to invest. In case they did not invest the €1, they would move to the next trial. In case they invested the €1, participants playing with humans and artificial races were told that their partners would receive €5 and in turn, decide either to cooperate, giving back €2.50 to the participant or to keep the whole money for themselves. Alternatively, participants playing with artworks were told that investing the €1 allowed them to enter the art market, which would reveal the real value of the painting up to €5. If the painting were of a high value, participants would earn the benefits of their investment shared with an art agency (i.e., €2.50). If the painting were of a low value, however, they would lose the invested €1. After making their decision, participants received visual feedback on their final outcomes (i.e., whether they received €2.50 or no money in return) during 1,000 ms. If participants did not respond within 1,500 ms, the message “You did not respond” appeared on the screen for 1,500 ms and concluded the trial. A 1,000 ms inter-trial black screen ended each trial.

The experiment comprised four phases: baseline, learning, discrimination test, and transfer. In the baseline phase (Block 1), participants were presented with 32 stimuli (i.e., 16 targets from Category 1 and 16 targets from Category 2). Each target was presented twice, once rewarding (i.e., associated with positive outcomes), and once non-rewarding (i.e., associated with negative outcomes), resulting in one block of 64 trials. The order of presentation of the trials was randomised independently for each participant.

In the following learning phase (Blocks 2–5), both categorical and individual information became relevant to predict the outcomes associated with a given target. The same targets used in the baseline were presented again, now probabilistically associated with positive or negative outcomes. We first established category–outcomes associations. Specifically, the two categories manipulated (e.g., men and women for the social dimension) were associated with opposite outcomes, either rewarding or non-rewarding. For instance, most men (12 out of 16) were cooperative and cooperated on 75% of the trials (i.e., men–rewarding association), while most women (12 out of 16) were noncooperative and cooperated only on 25% of the trials (i.e., women–non–rewarding association). The association between categories and rewards was counterbalanced across participants.

To examine the reliance on categorical or individuating information, we also established specific target–outcomes associations. Within each group, a small proportion of targets were reinforced in a way opposite to the category–outcomes associations, and are therefore referred to as inconsistent targets. Following the previous example, four inconsistent men were noncooperative and cooperated only on 25% of the trials, and four inconsistent women were cooperative and cooperated on 75% of the trials. Importantly, inconsistent partners were inconsistent with respect to the other members of their gender group but, at the individual level, continuously displayed the same cooperation rate in the learning phase (i.e., they cooperated on 75% or 25% of the trials).

Across the entire learning phase, each target was presented 16 times for a total of 512 trials divided into 4 blocks of 128 trials. Within each block, each target was presented four times. Rewarding targets were associated with positive outcomes on 75% of the trials (3 out of 4 trials) and with negative outcomes on 25% of the trials (1 out of 4 trials). Non-rewarding targets were associated with positive outcomes on only 25% of the trials (1 out of 4 trials) and negative outcomes on 75% of trials (3 out of 4 trials). The order of presentation of the trials was randomised within each block of 64 trials, independently for each block and each participant. The stimuli used to represent the inconsistent targets were counterbalanced such that across participants, all stimuli were associated with the inconsistent condition.

After the learning phase, participants performed a 5-min paper-pencil distraction task consisting of identifying the letter “Q” among arrays of letters “O” to delay the subsequent discrimination test. Next, they performed the discrimination test where they were presented with all the 32 stimuli from the learning phase and 32 new stimuli with whom they had no prior experience (16 from each category). Participants’ task was to indicate whether or not they had already been presented with those stimuli during the learning phase by pressing the “1” key if they recognised them, or the “0” key if they did not. In case they responded that they had been presented with a target stimulus, they were asked to indicate whether or not it was rewarding by pressing the “1” key if they thought it was, and the “0” if they thought it was not. This measure allowed us to analyse their recall of the stimuli presented in the trust game.^1^ The 32 fillers used in the discrimination test were presented as new individuals in the transfer phase. The order of presentation of the stimuli was randomised independently for each participant.

Finally, participants performed a transfer phase (Block 6) in which they were presented twice with 32 new targets that were not displayed in the previous baseline and learning phases, all of them being rewarding on one trial, and non-rewarding on the other trial. Therefore, and similarly to the baseline phase, neither categorical nor individual information was useful in predicting the outcomes associated with a target on a given trial. The transfer phase comprised a total of 64 trials, and the order of presentation of the trials was randomised independently for each participant.

At the end of the experiment, the experimenter extracted the percentage of accuracy of each participant from their data files and paid them immediately. The entire task lasted around 60 min. Participants were allowed auto-administered breaks every 64 trials.

### Analytic strategy

To examine whether participants were spontaneously biased to invest more with one of the two categories presented in the baseline, we compared the investment rate with the two categories of the same dimension (e.g., women vs. men). Hence, the baseline used a within-participants design with the variable Category analysed separately in the three dimensions (i.e., social dimension: men vs. women, social-like dimension: Lunaris vs. Taiyos, and non-social dimension: Cianelli vs. Kandinsky).

In the learning phase, we analysed whether participants’ learning of the reward value associated with consistent and inconsistent stimuli varied across dimensions. Hence, the learning phase used a mixed design with Dimension (social, social-like, non-social) as a between-participants factor and Consistency (consistent, inconsistent) and Blocks (2, 3, 4, 5) as within-participants variables. The DV was a learning index indicative of participants’ capacity to discriminate between rewarding and non-rewarding targets. Specifically, we subtracted investment rates with non-rewarding targets from investment rates with rewarding targets across the four blocks of trials, separately for consistent and inconsistent targets. Critically, this learning index in the inconsistent targets condition reveals whether participants adopted an individuating or categorising approach. Specifically, positive values indicate more investment with rewarding than non-rewarding targets, implying that participants discriminated rewarding from non-rewarding targets in their investment decisions. Conversely, negative values indicate more investment with non-rewarding than rewarding targets, that is, a categorisation pattern. Negative values were not expected for consistent targets.

In the transfer phase, we tested whether the learned category-outcomes associations established in the learning phase would impact participants’ investment decisions with new exemplars from these categories. A categorising approach of new targets would be reflected in more investment in the category that was rewarding in the learning phase. Hence, the critical comparison informing of whether participants adopted a categorical approach to the new targets was between the categories of the same dimension. The transfer phase used a mixed design with category as a within-participants variable, and Learned Rewarding Category (during the learning phase) as a between-participants factor, analysed separately in each dimension.

## Results

Data from all the participants were included in the analyses, whereas trials where participants did not make a timely decision (0.9%) or with reaction times faster than 200 ms (3.2%) were filtered out as in Tortosa, Lupiáñez, and Ruz (2013). The alpha criterion was set at .05. Following (Lakens, 2014), we computed 90% confidence intervals around partial eta-squared with the software provided by Nelson (2016), and 95% confidence interval around Cohen’s *d* with JASP 0.15 (JASP Team, 2022). For *p* values > .05, we reported BF_01_ using a *prior* Cauchy distribution (0.71) in JASP 0.15 (JASP Team, 2022). For within-subjects designs, we applied the Greenhouse–Geisser correction when the assumption of sphericity was violated.

### Baseline

Investment rates in the baseline were subjected to a within-participants analysis of variance (ANOVA) with Category (men vs. women, or Lunaris vs. Taiyos or Cianelli vs. Kandinsky) as a within participant variable, separately for each dimension (social, social-like, and non-social). The main effect of Category was significant in the group playing with humans (social dimension), *F*(1, 39) = 6.34, *p* = .016, $\eta_{\text{p}}^{2}$ = .14, 90% CI = [.01, .30], indicating that, as expected, participants invested more with female (*M* = .70, *SD* = .12) than with male (*M* = .64, *SD* = .15) partners. Hypothesis 1a was therefore supported. In contrast, participants did not significantly differ in their investment rates between Lunaris (*M* = .63, *SD* = .15) and Taiyos (*M* = .64, *SD* = .15), *F*(1, 39) = 0.09, *p* = .769, $\eta_{\text{p}}^{2}$ < .01, 90% CI = [.00, .07], BF_01_ = 5.62. Neither did they between artworks from Cianelli (*M* = .57, *SD* = .19) and Kandinsky (*M* = .64, *SD* = .18), *F*(1, 39) = 2.97, *p* = .093, $\eta_{\text{p}}^{2}$ = .07, 90% CI = [.00, .22], BF_01_ = 1.51, suggesting that Hypotheses 1b and 1c were also confirmed. In sum, only participants playing with humans were clearly biased to cooperate more with one of the two categories presented, and importantly, this expected pattern was in line with gender stereotypes (i.e., more cooperation with females than males).

### Learning

The learning index was subjected to a mixed-design ANOVA with Consistency (Consistent vs. Inconsistent), and Blocks (2, 3, 4, 5) as within-participants variables, and Dimension (social, social-like and non-social) as a between-participants factor.

**Figure 2.**
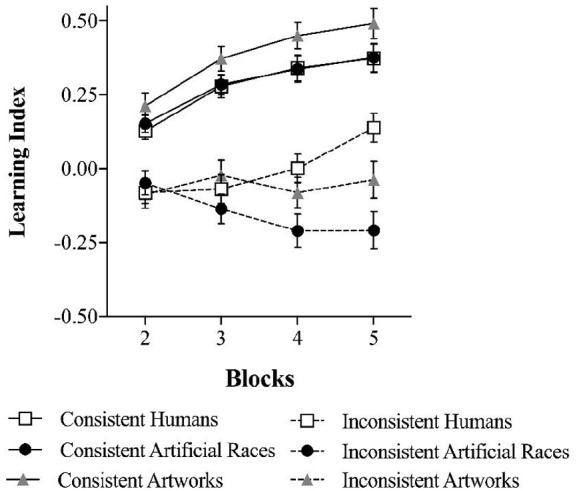
Learning about consistent and inconsistent exemplars across blocks in each experimental group. Error bars represent the standard error of the mean.

The expected Dimension × Consistency × Block interaction was significant, *F*(6, 351) = 2.80, *p* = .011, $\eta_{\text{p}}^{2}$ = .05, 90% CI = [.01, .07], indicating that the pattern of learning differed between the three experimental groups, as shown in Figure 2. To decompose this interaction, we first compared the three experimental groups’ learning about consistent targets before turning to learning about inconsistent targets.

In the consistent condition, the main effect of Block was significant, *F*(1.79, 209.60) = 52.11, *p* < .001, $\eta_{\text{p}}^{2}$ = .31, 90% CI = [.22, .38], and not qualified by Dimension, *F*(6, 351) = 0.26, *p* = .953, $\eta_{\text{p}}^{2}$ < .01, 90% CI = [.00, .00], indicating that all groups increasingly learned from Block 2 (*M* = .16, *SD* = .22) to Block 5 (*M* = .41, *SD* = .31), linear effect, *F*(1, 117) = 69.87, *p* < .001, $\eta_{\text{p}}^{2}$ = .37, 90% CI = [.26, .47], the outcomes associated with consistent targets, until reaching an asymptote, quadratic component, *F*(1, 117) = 26.80, *p* < .001, $\eta_{\text{p}}^{2}$ = .19, 90% CI = [.09, .29]. The main effect of Dimension was not significant, *F*(2, 117) = 2.53, *p* = .084, $\eta_{\text{p}}^{2}$ = .04, 90% CI = [.00, .10].

In the inconsistent condition, however, the Dimension × Block interaction was significant, *F*(6, 351) = 6.42, *p* < .001, $\eta_{\text{p}}^{2}$ = .10, 90% CI = [.04, .14], indicating that the three experimental groups learned to different extent the outcomes associated with inconsistent targets. Simple main effects analyses showed that participants playing with humans increasingly learned the outcomes associated with inconsistent partners from Block 2 (*M* = −.09, *SD* = .29) to Block 5 (*M* = .14, *SD* = .31), *F*(1, 39) = 16.64, *p* < .001, $\eta_{\text{p}}^{2}$ = .30, 90% CI = [.11, .46], suggesting, in line with Hypothesis 2a, an individuating approach. Participants playing with artificial races, however, showed a decreasing learning from Block 2 (*M* = −.05, *SD* = −.26) to Block 5 (*M* = −.21, *SD* = .40), *F*(1, 39) = 7.84, *p* = .008, $\eta_{\text{p}}^{2}$ = .17, 90% CI = [.02, .32], suggesting, as predicted by Hypothesis 2b, a larger reliance on categorical information, and therefore an impaired learning of the information inconsistent with the categorical information. Finally, for participants playing with artworks, learning about inconsistent target neither increased nor decreased, *F*(1, 39) = 0.25, *p* = .623, $\eta_{\text{p}}^{2}$ < .01, 90% CI = [.00, .14], BF_01_ = 3.98, remaining steady from Block 2 (*M* = -.09, *SD* = .29) to Block 5 (*M* = −.04, *SD* = .40), suggesting a more moderate approach but an impaired learning of the target–outcome associations inconsistent with the category–outcomes associations, supporting Hypothesis 2c.

Importantly, in Block 5, learning scores about inconsistent targets were significantly above 0 for humans, *t*(39) = 2.85, *p* = .007, *d* = 0.45, 95% CI = [0.12, 0.77], significantly below 0 for artificial races, *t*(39) = −3.31, *p* = .002, *d* = −0.53, 95% CI = [−0.85, -0.19], and not different from 0 for artworks, *t*(39) = −0.59, *p* = .557, *d* = −0.09, 95% CI = [−0.40, 0.22], indicating that at the end of the learning phase, only human targets were individuated, while artificial races were categorised, and artworks fell in between these two opposite approaches. One-tail Bayesian *t*-tests supported these observations, with BF_0+_ = 8.73 for artworks and BF_0+_ = 22.67 for artificial races, suggesting that the absence of learning about inconsistent targets were, respectively, 8.73 and 22.67 times more likely than learning about them. Although the learning index for artworks was higher compared with the artificial races, this difference did not reach statistical significance, *t*(78) = 1.93, *p* = .057, *d* = 0.43, 95% CI = [0.01, 0.87], and Bayesian evidence was inconclusive, BF_01_ = 0.87. Importantly, however, this pattern was reversed in the case of humans, BF_0+_ = 11.02, in which learning about inconsistent targets were around 11 times more likely than non-learning.

In sum, only participants playing with humans learned across blocks whether or not to invest with both consistent and inconsistent exemplars, whereas only participants playing with artificial races clearly categorised inconsistent exemplars.

### Discrimination test

To have a sense of participants’ capacity of discrimination between the targets presented in the trust game and new stimuli, we followed the signal detection theory (Stanislaw & Todorov, 1999) to analyse their performance in the discrimination test. Specifically, we considered as hits trials those in which participants accurately identified that the face (or artwork) presented was also presented in the trust game, and false alarms those trials in which participants mistakenly responded that the face (or artwork) presented was also presented in the trust game when it was not. From these trials, we extracted a sensitivity index (*d*′) reflecting participants’ capacity to discriminate between stimuli, such that higher *d*′ values indicated a higher discrimination capacity. We observed that all participants discriminated new exemplars from exemplars presented in the trust game above chance in the groups playing with humans (*M* = 3.67, *SD* = 0.11), *t*(39) = 31.57, *p* < .001, *d* = 4.99, 95% CI = [3.84, 6.11], artificial races (*M* = 0.75, *SD* = 0.14), *t*(39) = 5.57, *p* < .001, *d* = 0.88, 95% CI = [0.51, 1.24], and artworks (*M* = 2.52, *SD* = 0.10), *t*(39) = 25.56, *p* < .001 *d* = 4.04, 95% CI = [3.09, 4.98]. Moreover, a univariate ANOVA revealed a main effect of Dimension, *F*(2, 117) = 155.13, *p* < .001, $\eta_{\text{p}}^{2}$ = .73, 95% CI = [.65, .77]. Post hoc analyses with Bonferroni correction showed that humans were better discriminated than artworks, *t*(78) = 6.89, *p* < .001, and artificial races *t*(78) = 17.48, *p* < .001. Artworks were also better discriminated than artificial races, *t*(78) = 10.60, *p* < .001.

**Figure 3.**
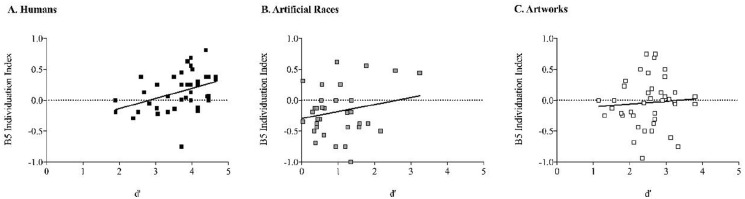
Correlation between *d*′ and the individuation index in the last block of learning.

We further explored the relationship between participants’ capacity of discrimination and their learning about inconsistent individuals in the trust game. Specifically, we ran a bivariate correlation analysis with the sensitivity index (*d*′) and the learning index about inconsistent individuals in Block 5, when learning was well established. Considering all the sample, we observed that discrimination capacity was positively correlated with learning from inconsistent individuals, *r*(120) = .42, *p* < .001. That is, the better participants discriminated the targets, the more they learned about their individual cooperative behaviours in the trust game. However, further analyses in each experimental group (see Figure 3) suggest that not all groups took advantage of their capacity of discrimination to individuate inconsistent targets. The group playing with humans was the only one to show a significant positive correlation, *r*(40) = .39, *p* = .012. A similar trend was observed in the group playing with artificial races, albeit the correlation did not reach statistical significance, *r*(40) = .24, *p* = .132, [BF_01_ = 1.70]. However, there was no hint of a correlation between discrimination capacity and learning in the group playing with artworks, *r*(40) = .07, *p* = .661, [BF_01_ = 4.66]. That is, in this group, a better capacity to discriminate between exemplars did not result in a larger learning, as shown in Figure 3. Therefore, despite the general tendency of individuation positively correlating with discrimination, only participants playing with humans (and to some extent with artificial races) translated their discrimination skills into more individuation.

### Transfer phase

Investment rates in the transfer phase were subjected to mixed-design ANOVAs with Learned Rewarding Category during the learning phase (men vs. women, or Lunaris vs. Taiyos or Cianelli vs. Kandinsky) as a between-participants factor (e.g., for half of the participants playing with humans, men were the rewarding category while for the other half of the participants playing with humans, women were the rewarding category), and Target (men vs. women, or Lunaris vs. Taiyos or Cianelli vs. Kandinsky) as a within-participants variable, separately for each dimension.

**Figure 4.**
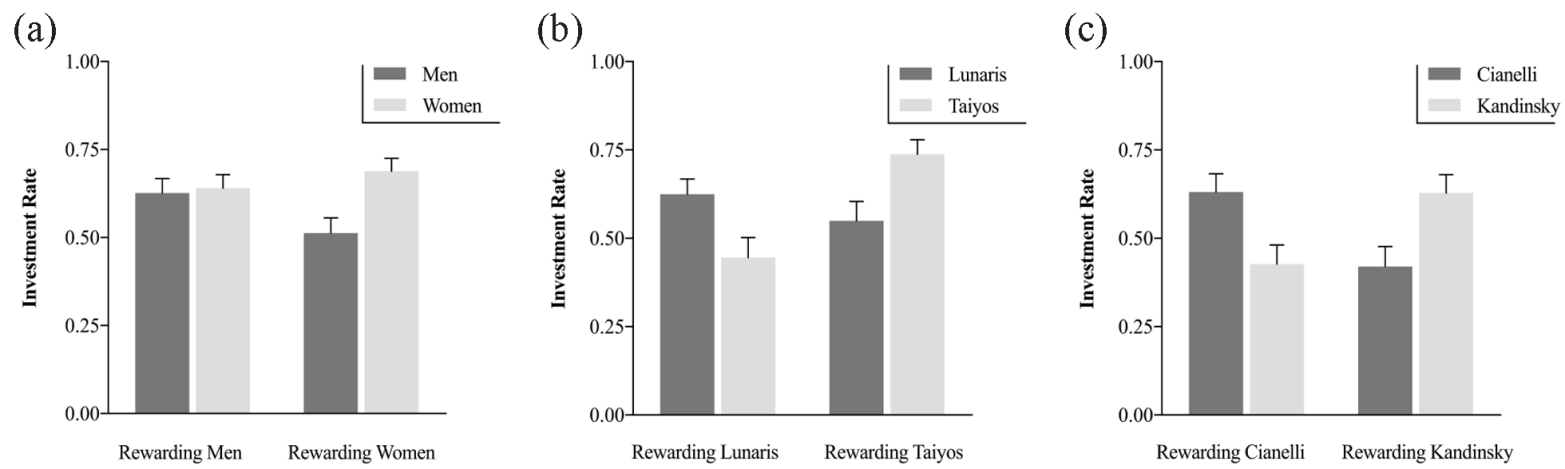
Investment with new targets in the transfer phase according to the rewarding category in the learning phase for the groups playing with (a) humans, (b) artificial races, and (c) artworks. Error bars represent the standard error of the mean.

As shown in Figure 4, in the group playing with humans, the main effect of Target was significant, indicating that participants invested more with women (*M* = .66, *SD* = .17) than with men (*M* = .58, *SD* = .19), *F*(1, 38) = 7.67, *p* = .009, $\eta_{\text{p}}^{2}$ = .17, 90% CI = [.03, .33]. Moreover, the critical Target x Learned Rewarding Category interaction was significant, *F*(1, 38) = 34.92, *p* < .001, $\eta_{\text{p}}^{2}$ = .47, 90% CI = [.27, .61]. Simple main effects analyses showed that when women were the rewarding group in the learning phase, participants invested more with new women (*M* = .69, *SD* = .16) than with new men (*M* = .51, *SD* = .19) in the transfer phase, *F*(1, 19) = 11.61, *p* = .003, $\eta_{\text{p}}^{2}$ = .38, 90% CI = [.10, .57]. However, when in the learning phase men were the rewarding group, participants did not significantly differ in their investment between men (*M* = .63, *SD* = .18) and women (*M* = .64, *SD* = .17), *F*(1, 19) = .26, *p* = .614, $\eta_{\text{p}}^{2}$ = .01, 90% CI = [.00 .17], BF_01_ = 3.82.

In the group playing with artificial races, the Target × Learned Rewarding Category interaction was also significant, *F*(1, 38) = 16.20, *p* < .001, $\eta_{\text{p}}^{2}$ = .30, 90% CI = [.11, .46]. Simple main effects analyses showed that when in the learning phase Lunaris were the rewarding group, participants invested more with new Lunaris (*M* = .63, *SD* = .19) than with new Taiyos (*M* = .45, *SD* = .25), *F*(1, 19) = 7.58, *p* = .01, $\eta_{\text{p}}^{2}$ = .29, 90% CI = [.04, .49]. When in the learning phase Taiyos were the rewarding group, participants invested more with new Taiyos (*M* = .74, *SD* = .18) than with new Lunaris (*M* = .55, *SD* = .24), *F*(1, 19) = 8.64, *p* = .008, $\eta_{\text{p}}^{2}$ = .31, 90% CI = [.05, .52].

A similar pattern was observed in the group playing with artworks. The Target × Learned Rewarding Category interaction was significant, *F*(1, 38) = 11.24, *p* = .002, $\eta_{\text{p}}^{2}$ = .23, 90% CI = [.06, .39]. Simple main effects analyses showed that when in the learning phase Cianelli’s paintings were rewarding, participants invested more with new Cianelli’s (*M* = .63, *SD* = .23) than with new Kandinsky’s (*M* = .43, *SD* = .24) artworks, *F*(1, 19) = 5.41, *p* = .031, $\eta_{\text{p}}^{2}$ = .22, 90% CI = [.01, .44]. Similarly, when in the learning phase Kandinsky’s artworks were rewarding, participants invested more in new Kandinsky’s (*M* = .63, *SD* = .23) than in new Cianelli’s (*M* = .42, *SD* = .25) artworks, *F*(1, 19) = 5.83, *p* = .026, $\eta_{\text{p}}^{2}$ = .24, 90% CI = [.02, .45]. As shown in Figure 4, cooperation rates were perfectly symmetrical across the two categories showing that after learning, participants based their investment decisions on the acquired categorial information.

In sum, the category–outcomes associations learned during the learning phase were generalised to new exemplars, especially in the case of artificial races and artworks, supporting Hypotheses 3b and 3c, respectively. In the case of social stimuli, Hypothesis 3a was partially supported, since the categorisation pattern was observed when women (but not men) were the rewarding category, suggesting an impact of gender stereotypes in the transfer phase.

## Discussion

The present research investigated the reliance on categorical versus individuating information when making investment decisions with social, social-like, and non-social targets. Overall, the data from the three phases of the trust game support the hypothesis that investment decisions with social-like (i.e., artificial races) and non-social stimuli (i.e., artworks) are mainly guided by categorial processing, while social stimuli (i.e., humans) are flexibly categorised or individuated depending on prior information.

In first interactions with unknown individuals (baseline phase), participants playing with humans were biased when inferring the outcomes associated with male and female targets, investing more with female than with male partners. This pattern is consistent with gender stereotypes associating women with more cooperation than men (Buchan et al., 2008; Cuddy et al., 2008), but may also reflect—especially in a sample comprising a large majority of female participants (i.e., 34 out of 40)—an ingroup bias, that is, a tendency to favour own- as opposed to out-group members (Balliet et al., 2014; Turner et al., 1979). Either because of gender stereotypes or ingroup bias, this pattern reveals that participants’ cooperation pattern in the baseline was biased by their beliefs or motivational concerns prior to the trust game. In contrast, participants playing with artificial races and artworks did not favour any of the two categories presented. Therefore, in first interactions, impressions about previously unknown social stimuli were primarily underlain by categorisation processes in the form of stereotypes or ingroup bias, while impressions about social-like and non-social stimuli were unbiased due to the lack of prior associations between the categories used in this experiment and specific reward patterns.

Across repeated interactions (learning phase), participants playing with humans abandoned stereotypes and updated their impressions based on the individuating information acquired during the game (Chang et al., 2010; Telga et al., 2018). In contrast, participants playing with non-social stimuli (artificial races and artworks) learned to rely on the categorical information acquired across trials to make investment decisions, but learned much less about the target–outcomes associations inconsistent with the well-learned categorical information, thus showing less individuation.

This categorical tendency was confirmed in the subsequent transfer phase for participants playing with non-social stimuli. When investing in newly presented artificial races and artworks, participants showed a pattern of responses that resemble member-to-member generalisation (Ranganath & Nosek, 2008; Vermue et al., 2019), basing their decisions on the category–outcomes associations established in the learning phase. Participants playing with humans, however, were more moderate and only applied the knowledge acquired in the learning phase when it was consistent with pre-existing beliefs on men and women cooperativeness (i.e., when women were more cooperative and men less cooperative in the learning phase). Although consistent with previous research showing member-to-member generalisation in the Trust Game (Vermue et al., 2019), our results are also different in that they suggest that this type of categorisation may interact with prior beliefs on the members of a social category. In sum, participants playing with social-like and non-social stimuli increasingly relied on categorical information as the task progressed. In contrast, participants who played with humans only used categorical information when deemed coherent with their own beliefs (or in the absence of any information, as in the baseline).

Although this experiment was not initially designed with this purpose, it might be insightful to consider the data reported here according to the two main models of reinforcement learning (RL): model-free versus model-based RL. In both models, the agent aims to maximise their reward (e.g., maximise their economic reward in a trust game) and needs to adjust their behaviour to the environmental contingencies (Sutton & Barto, 1998). One of the critical differences between the two models lies in the flexibility to update this behaviour. In a model-free RL, learning is constrained by the acquired instrumental relationship and, consequently, such behaviour is less adaptable to the environment. For example, an agent may persist in repeating an acquired response (at least until some point), even though this action is no longer associated with the expected outcome (Daw et al., 2011). In a model-based RL, however, the agent is more capable of considering additional environmental information and learning is not merely driven by action–outcome associations. The agent is, therefore, more capable of adapting to a changing environment. Depending on the environmental settings, the agent shows a preference for one type of pattern (Daw et al., 2011), although there is a large individual variance (see Hackel et al., 2019).

Given its structure resembling a changing environment (i.e., varying category– and target–outcome associations across the three phases), our trust game might capture to some extent different types of reinforcement learning models. In fact, the pattern of results observed with participants playing with social-like and non-social stimuli fits a model-free approach: once learning was established (learning phase), participants kept applying the category-outcome associations learned even in a different context (i.e., new targets in the transfer phase), when these associations were no longer relevant. In contrast, in the case of participants playing with humans, the approach was more mixed, as they showed a pattern of data coherent with a model-free learning when the category–outcomes associations learned were consistent with gender stereotypes, but a model-based approach when the contingencies learned in the learning phase challenged their prior beliefs about the relationship between gender and cooperation.

Interestingly, the groups showing more categorisation (i.e., participants playing with non-social and social-like stimuli), adopted a model-free approach in the transfer phase, presumably because the only information they had about the targets was the one learned in the learning phase of the trust game. However, participants playing with humans could rely on both gender stereotypes and the category–outcomes associations from the trust game, and adopted a moderate approach neither entirely model-based nor completely model-free. These data fit the idea that in the absence of informative contingencies (such as baseline or transfer phase where the category–outcome associations were random), participants engaged in high-order processes, incorporating information from outside the instrumental contingencies (Kurdi et al., 2019). The observed interplay between social (top-down processes, e.g., stereotypes) and non-social (bottom-up processes, e.g., action-outcome contingency) information fits the idea that these two sources of information are bidirectionally and continuously affecting one another, in line with the hierarchical architecture of social learning (Otten et al., 2017).

Despite the overall pattern of results indicated that each group applied a specific pattern of behaviour in each phase, the differences observed may also be accounted for by other factors not directly related with the “socialness” of the stimuli. Because participants discriminated better humans than artworks, and discriminated better among artworks than among artificial races, the findings from the present research may reflect participants’ perceptual expertise. That is, they may have showed a better performance with humans compared with artificial races and artworks because they are more skilled at discriminating humans than artificial races and artworks. However, the fact that participants’ capacity of discrimination did not always correlate with the magnitude of individual learning observed suggests that perceptual expertise alone is not sufficient to explain the patterns of data. In fact, the discrimination scores for artworks were excellent and fairly close to the discrimination scores observed with humans. At any rate, the data relative to *d*′ during the discrimination test support a good discrimination (clearly above chance) in all groups. However, in contrast to humans, artworks were categorised both in the learning and the transfer phases, suggesting that when playing with artworks, participants did not fully take advantage of their capacity of discrimination to make accurate decisions about artworks.

It should also be noted that in the present study, targets were presented for a very brief period of time, forcing participants to make their decision under time pressure (equally for all groups). This methodological approach has been largely used in previous research (Telga et al., 2018; Telga & Lupiáñez, 2021; Tortosa, Lupiáñez, & Ruz., 2013; Tortosa, Strizhko, et al., 2013) and allowed us to limit the duration of the study, but may have impacted the results. In fact, Hughes et al. (2017) showed that time pressure reduces cognitive control, resulting in more categorical bias (e.g., ingroup bias). Therefore, the experimental procedure used here may have promoted the reliance on categorical information, particularly with non-social stimuli with whom participants were less familiar. Further research is needed to assess whether, without time pressure, the reliance on categorical processes would be reduced and with it, the between-group differences observed in the present study.

It is also worth considering that artificial races arguably resemble male (compared with female) faces, which may have activated gender stereotypes in the experimental group playing with these stimuli. Because the large majority of participants in this experiment were women, any gender-related effect could also be understood in terms of intergroup biases. If participants readily associated artificial races with male faces, they may have activated gender processes similar to what would be expected with human male faces. Given that in iterative trust games, outgroup may be more categorised than ingroup members (Telga et al., 2018), it is possible that the masculinity of the artificial races also contributed to the reliance on categorisation processes in this experimental group. Future research should test this possibility.

Finally, despite we used the same structure and pay-offs for the trust game in the three experimental groups, the framing was slightly different. In fact, the trust game was framed as an economic game about investment in art in the non-social group, while more emphasis was put on cooperation in the two other groups. Therefore, the social component of a framing based on cooperation in the social and social-like groups may have added an extra layer of “socialness” in these groups. The key manipulation of the present experiment was to create a clear distinction between social and non-social stimuli, something achieved through both the framing of the trust game and the physical features of the stimuli. In future studies, it would be interesting to test separately the effect of the framing on one hand, and the physical features of the stimuli on the other hand, to assess whether both dimensions equally contribute the observed effects.

Although it seems clear that social stimuli are more likely to promote an individuating approach than social-like and non-social stimuli, the fact that the three experimental groups were not evenly matched in discrimination ability makes the interpretation of the data complex. Some methodological improvements in future studies may help to disentangle the role of motivation and cognitive ability in the differentiated use of individuation and categorisation strategies. An ideal starting point would be to match the three experimental groups on discrimination and categorisation ability before the trust game, which may not be realistic because of the necessary varying degrees of familiarity with the stimuli across dimensions. To circumvent this issue, an alternative would be to use the same stimuli across conditions but manipulating participants’ beliefs about whether they are interacting with either social or non-social agents (see Hackel et al., 2015, 2020 for a similar procedure).

Despite these limitations, we believe that the protocol used in the present research is an excellent versatile tool to explore the impact of categorical and individuation information on economic decision-making. Specifically, with its three phases, it captures biases that may emerge in social interactions at zero acquaintance (e.g., stereotypes, ingroup bias), as well as the more subtle influence of categorical information across repeated interactions (see also Telga et al., 2018), or after learning specific category–outcomes associations (Vermue et al., 2019). In addition, it may be used to explore not only trust relationships between people, but also investment decisions with non-social stimuli. It may as well be used to investigate the emergence of categorical processes with a large range of stimuli, a topic of particular relevance for research investigating, for instance, exemplar-based categorisation (Murphy, 2010; Nosofsky, 1984). Because this protocol allows researchers to manipulate a series of social (e.g., specific social groups, expectations) and cognitive (e.g., attentional resources, perceptual expertise) factors relevant in impression formation processes (Fiske & Neuberg, 1990), it is a valuable tool to explore a large range of psychological processes and to bridge the social, cognitive, and economic psychology literature.

Finally, understanding trust decisions with non-human agents is becoming a major challenge for both economists and psychologists. As technological advances increasingly involve interactions with artificial agents such as web bots customer support, or programmed assistance for medical advice, we, as human agents, often have to deposit our trust in non-social agents (Andras et al., 2018; Han et al., 2021). Given the growing importance of the interaction between human and non-social agents, it is necessary to understand to what extent the well-studied psychological phenomena that underly social interactions also underly our interactions with non-social agents. With this experiment, we hope to contribute to the growing body of research aiming to understand the commonalities and discrepancies between social and non-social interactions.

## Conclusion

The current research provides novel evidence that social and non-social stimuli trigger different behavioural processes. Although people primarily rely on resources-saving categorical information to make investment decisions, they are more likely to adopt an individuating approach with humans compared with non-social stimuli. At a time when machine–human interactions are more and more frequent, understanding how humans approach social and non-social interactions becomes crucial.

## Supplemental Material

sj-docx-1-qjp-10.1177_17470218231153137 – Supplemental material for Social and non-social categorisation in investment decisions and learning Click here for additional data file. Supplemental material, sj-docx-1-qjp-10.1177_17470218231153137 for Social and non-social categorisation in investment decisions and learning by Maïka Telga, José A Alcalá and Juan Lupiáñez in Quarterly Journal of Experimental Psychology

## Notes

1. The analyses of this test are provided in the second section of the Online Supplementary Material.
