# Supplementary material for: Social and non-social categorisation in investment decisions and learning
Source: Q J Exp Psychol (Hove). 2023 Feb 4;76(12):2718–31. doi: 10.1177/17470218231153137 (PMC10655696; doi:10.1177/17470218231153137)
Supplement: sj-docx-1-qjp-10.1177_17470218231153137 – Supplemental material for Social and non-social categorisation in investment decisions and learning [file sj-docx-1-qjp-10.1177_17470218231153137.docx]

Supplementary Material for:

**Social and non-social categorization in investment decisions and learning.**

Maïka Telga^1,2^, José A. Alcalá^3,4^, & Juan Lupiáñez^1^

^1^University of Granada

^2^University of St Andrews

^3^University of Jaén

^4^ University Complutense of Madrid

## 1. Experimental instructions [translated from Spanish]

### 1.1. Verbal instructions provided to all participants

“Thank you for coming in and taking part in the experiment. If you have a cellphone, please turn it off or on silent. You are going to complete a study on economic decision-making. At the beginning of each trial, you will virtually receive €1. Across the trials, you will be presented with several stimuli and will have to decide what to do with the money received. Your decision in each trial can lead you to either monetary gains or losses. You will be provided with more specific instructions about the structure of the task and the exact payoffs on the computer screen before you start. The task depicts fictitious scenarios. This means that the associations between the stimuli and economic values are not real, and you are not playing online. But please, try to immerse yourself as much as possible in the task and do it to the best of your capacity. Depending on your performance, you can earn up to €10. For example, if you respond correctly on 50% of the trials, you will earn €5, if you do on 70% of the trials, you will earn €7, etc. The task is quite long but you will have the opportunity to take a break on several occasions. When you are done with the study or if you have questions at any time, just come out and get me. Do you have any question before you start?”

### 1.2. Visual instructions

#### 1.2.1. Instructions displayed to participants playing with artificial races and humans

This study explores decision-making in economic contexts. You will realize this task with different game partners. At the beginning of each round, you will receive €1 and you will have to decide whether:

- You want to cooperate with your partner in this round by sending them your €1, or
- You want to keep the €1 for yourself and do not want to cooperate.

If you decide to send your €1, your partner will receive €5. Then, your partner will decide to share those €5 with you or not. You will be informed of your partner’s decision in each round. In each round, your game partner(s) will decide between:

- Cooperate, in this case they will give you back half of the money, €2.50, or
- Not cooperate, in this case they will keep the €5 and will give you nothing

If you decide not to cooperate and to keep the €1 at the beginning of the round, you will win this €1 and your game partner(s) will receive nothing in this round.

These are the possible outcomes in each round:


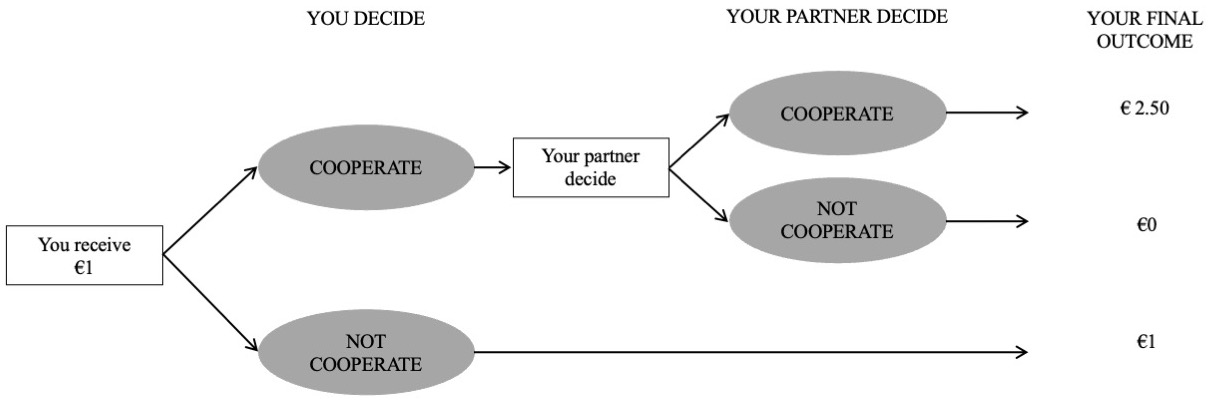


Your goal is to maximize your benefits for the entire game. The best strategy to achieve this goal is to cooperate with partners who share the money with you, and to not cooperate with partners who do not to share the money with you. You will have several trials with all partners to determine their tendency to share.

At the beginning of each round, you will receive €1. Next, a fixation point (+) will appear in the center of the screen. Please focus on this point. Your partner in this round will appear for 1 second. From the moment the question appears, you will have 1,5 seconds to respond.

If you decide to **SEND** your €1, press the **'1'** key.

If you decide to **KEEP** your €1, press the **'0'** key.

Remember that your goal is to maximize your benefits with each partner. Also, you will be rewarded with real money proportionally to the amount won during the task.

PRESS THE SPACEBAR TO START

#### 1.2.2. Instructions displayed to participants playing with artworks

This study explores decision-making in economic contexts. You will realize this task with different artworks. At the beginning of each round, you will receive €1 and you will have to decide whether:

- You want to invest your €1 in the artwork of this round, or
- You want to keep the €1 for yourself and do not invest anything

If you decide to send your €1, you will access the art market whose fluctuations will reveal the real value of the artwork, and your investment may be converted into €5. Therefore, you may earn more money thanks to your investment, or lose your investment if the artwork is not worth €5. You will be informed of the real value of the artwork in each round. The outcomes of your investment may be:

- Positive, in this case you will receive half of the value of the artwork, €5, or
- Negative, in this case you will lose the invested money and receive nothing

If you decide not to invest and to keep the €1 at the beginning of the round, you will win this €1 and will not enter the art market in this round.

These are the possible outcomes in each round:


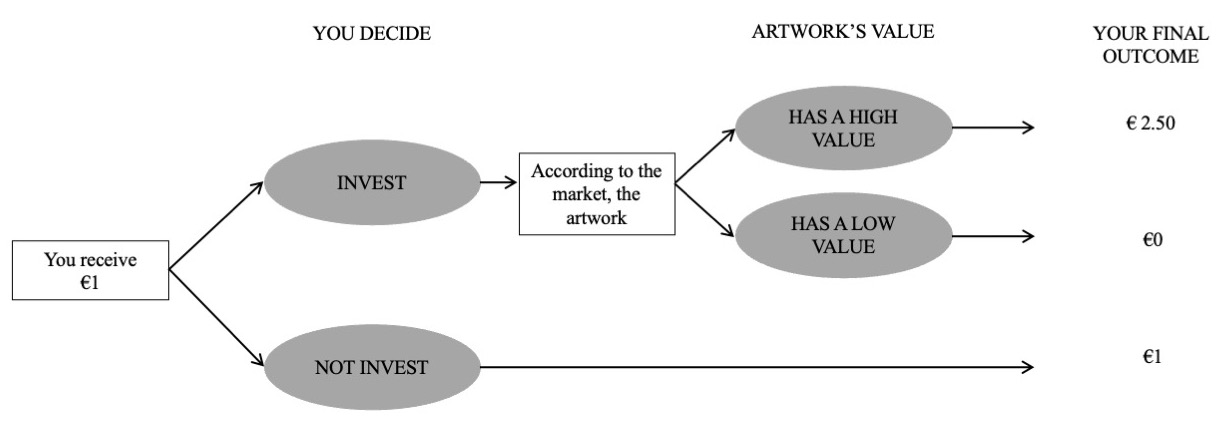


Your goal is to maximize your benefits for the entire game. The best strategy to achieve this goal is to invest in the artworks that tend to be positively revaluated, and not to invest in the artworks that tend to be negatively revaluated.

At the beginning of each round, you will receive €1. Next, a fixation point (+) will appear in the center of the screen. Please focus on this point. The artwork of this round will appear for 1 second. From the moment the question appears, you will have 1,5 seconds to respond.

If you decide **to** **INVEST** your €1, press the **'1'** key.

If you decide **NOT to INVEST** your €1, press the **'0'** key.

Remember that your goal is to maximize your benefits with each artwork. Also, you will be rewarded with real money proportionally to the amount won during the task.

PRESS THE SPACEBAR TO START

## 2. Investment decisions in the trust game

|  | Invest | Did not invest | Did not respond in time |
| --- | --- | --- | --- |
| Humans | 59.24 | 39.90 | 0.86 |
| Artificial races | 59.51 | 39.50 | 0.99 |
| Artworks | 54.03 | 45.06 | 0.91 |
| Total | 57.59 | 41.49 | 0.92 |

**Table 1:** Percentage of trials where participants invested, did not invest or did not make a timely decision.

## 3. Recall results from the discrimination test

A different repeated-measures ANOVA was conducted on recall scores with Reward (Rewarding vs. Non-rewarding) and Consistency (Consistent vs. Inconsistent) as within-participants variables in each experimental group.

In the group playing with humans (social dimension), we found a significant main effect of consistency, *F*(1, 37) = 24.77, *p* < .001, $ƞ_{p}^{2}$ = .40, 90% CI = [.19, .55], indicating that participants recalled better the cooperative behaviors of consistent (*M* = .78, *SD* = .13) than inconsistent (*M* = .61, *SD* = .20) partners.

In the group playing with artificial races (social-like dimension), the main effect of consistency was also significant, *F*(1, 34) = 33.14, *p* < .001, $ƞ_{p}^{2}$ = .49, 90% CI = [.28, .62], and qualified by the Reward variable, *F*(1, 34) = 4.81, *p* = .035, $ƞ_{p}^{2}$ = .12, 90% CI = [.00, .29]. Participants recalled better consistent than inconsistent partners both within the rewarding category, *F*(1, 37) = 11.87, *p* = .001, $ƞ_{p}^{2}$ = .24, 90% CI = [.07, .41], and the non-rewarding category, *F*(1, 34) = 47.51, *p* < .001, $ƞ_{p}^{2}$ = .58, 90% CI = [.38, .69], although the difference between consistent and inconsistent was larger in the non-rewarding category (*M* = .77, *SD* = .23 vs. *M* = .28, *SD* = .31) than in the rewarding category (*M* = .74, *SD* = .21 vs. *M* = .44, *SD* = .40).

A similar pattern of data was observed in the group playing with artworks (nonsocial dimension). The main effect of consistency was significant, *F*(1, 39) = 14.03, *p* < .001, $ƞ_{p}^{2}$ = .27, 90% CI = [.08, .43], and qualified by the Reward variable, *F*(1, 39) = 7.93, *p* = .008, $ƞ_{p}^{2}$ = .17, 90% CI = [.03, .33]. Participants significantly recalled better consistent (*M* = .82, *SD* = .24) than inconsistent (*M* = .51, *SD* = .31) artworks within the non-rewarding category, *F*(1, 39) = 22.71, *p* < .001, $ƞ_{p}^{2}$ = .37, 90% CI = [.17, .52]. However, in the rewarding category, participants showed similar recall of consistent (*M* = .72, *SD* = .19) and inconsistent (*M* = .61, *SD* = .34) artworks, *F*(1, 39) = 2.67, *p* = .110, $ƞ_{p}^{2}$ = .06, 90% CI = [.00, .21].

In summary, participants recalled better the outcomes associated with consistent than with inconsistent targets. However, this pattern seemed clearer within the non-rewarding category, especially for the social-like and non-social dimensions.

## 4. Pilot Studies

Different experiments were conducted to test the stimuli to be used as targets in the trust game. Importantly, we aimed at verifying that within each dimension (i.e., humans, artificial races, paintings), participants would be able to categorize the targets into the two categories of interest, and to discriminate among the exemplars of each category. Here, we report the results for the stimuli that were finally selected for the trust game.

### 4.1. Method

4.1.1. Participants. Across the pilot studies, different groups of participants evaluated different sets of stimuli. The data reported in the present research were collected in 3 different experiments, either testing stimuli for the human, the artificial races or the painting dimensions. Twenty volunteers participated in each one of these experiments in exchange for a credit course. Written informed consents were obtained from all participants and the experiments were conducted following the guidelines approved by the local ethic committee (175/CEIH/2017).

4.1.2. Apparatus and stimuli. Stimuli were displayed on a 27’’ screen placed at 60 cm from participants. E-Prime 2.0 software (Schneider et al., 2002) was used for stimuli presentation and data acquisition. For the social condition, we tested participants’ categorization based on gender groups: men and women. We used the pictures of 32 men and 32 women against a grey background with neutral facial expressions taken from The Karolinska Directed Emotional Faces (KDEF) (Lundqvist et al., 1998). For the social-like condition, we verified that participants distinguished between two artificial races created in the lab with human-like attributes: Lunaris and Taiyos. In fact, research has shown that people can learn to individuate Lunaris and Taiyos and may process artificial races’ faces holistically, just as human faces (Chua et al., 2014). Thirty-two pictures of Lunaris and 32 pictures of Taiyos with bottom and top variations were ceded by the Object Perception Lab of Vanderbilt University. Finally, for the non-social condition, we expected participants to distinguish paintings from two artists in a categorical way, as observed in previous studies (dos Santos Ferreira et al., 2018). In fact, paintings are fairly complex stimuli and allow to build a credible cover story for the trust game. After various pilot studies testing paintings from different artists, we finally selected 32 paintings from Wassily Kandinsky and 32 paintings from Jaison Cianelli, with unequivocally different styles. While Kandinsky’s paintings were characterized by the use of overlapping geometrical figures, Cianelli’s paintings rather consisted of shapeless melted colors. Kandinsky’s paintings were obtained via a Google search while Cianelli’s paintings were downloaded from the artist’s website. All the selected paintings were abstract to prevent participants from associating the contents of the paintings with social components. The pictures of humans and artificial races were displayed at 8.10º x 10.95º, with a total area of 88.70º. The paintings were resized such that their area was as close as possible to 88.70º, while maintaining their original shape.

4.1.3. Procedure. Participants performed the task with either humans (men and women), artificial races (Lunaris and Taiyos) or paintings (Cianelli’s and Kandinsky’s). First, they completed a categorization task in which they had to classify 32 stimuli (i.e., 16 exemplars from each category) into the categories of interest. Visual instructions about the categorization task included one example for each category without a label or any explicit mention of the dimensions of categorization manipulated. Participants first performed 8 practice trials consisting of 2 presentations of 4 exemplars from each category (different from the 32 stimuli of the experimental block), in which they received visual feedback. Afterwards, they started the experimental phase consisting of 6 presentations of the 32 stimuli. Each trial started with a fixation cross displayed for 1 second followed by the picture of an exemplar from one of the categories manipulated for 1.5 seconds or until response. No feedback was provided during the experimental phase. The inter-trial interval was 1 second. Auto-administered breaks were allowed every 64 trials. Upon the completion of the categorization task, participants performed a memory test in which they were presented with the 32 stimuli from the categorization task and 32 new stimuli from the same categories. Each stimulus was displayed for 1.5 seconds and participants had to indicate whether or not the picture had been presented during the categorization phase. Overall, the experiment lasted around 35 minutes.

### 4.2. Results and discussion

To test whether participants equally categorize the exemplars from the two categories manipulated in each dimension, the accuracy scores in the categorization task were subjected to separate repeated-measures ANOVAs with the target (men vs. women, Lunari vs. Taiyo, or Cianelli vs. Kandinsky) as a within-participants variable. This analysis revealed that within each experiment, participants did not significantly differ in their categorization of the two targets of interest, larger *F*(1, 19) = 1.65, *p* = .215, $ƞ_{p}^{2}$ = .08, 90% CI = [.00, .29], in the experiment with paintings. Further, to verify whether participants categorize the targets above chance level, the same accuracy scores were subjected to a one-sample T-test, revealing that exemplars from all categories were categorized above chance, smaller *t*(19) = 6.50, *p* < .001, for men categorization. Overall, these data revealed that for each dimension, the targets were accurately categorized in the two categories of interest, and that the process of categorization was similar for both categories.

To test whether participants equally discriminated the targets within the categories of interest, accuracy scores from the memory test were subjected to a separate repeated-measures ANOVA with the target as within-participants variable. Again, in all experiments, participants did not significantly differ in their recognition of the targets from the two categories manipulated, larger *F*(1, 19) = 2.90, *p* = .105, $ƞ_{p}^{2}$ = .13, 90% CI = [.00, .35], for participants playing with paintings. We also verified that participants discriminated the targets from all categories above chance. A one-sample T-test on accuracy in the memory test confirmed that targets from all categories were recognized above chance, smaller *t*(19) = 3.67, *p* < .002, for Lunaris’ recognition. Overall, these data suggested that participants discriminated the targets accurately, and that discrimination was similar for the two categories of interest within each dimension.

Between-experiments analyses were conducted with separate one-way ANOVAs on memory and categorization scores. Dimension (humans vs. artificial races vs. paintings) was used as a between-participants factor, to have some sense of potential between-group differences in categorization and discrimination capacity. This analysis revealed a significant effect of dimension on memory scores, *F*(2, 57) = 117.65, *p* < .001, $ƞ_{p}^{2}$ = .81, 90% CI = [.72, .85]. Post-hoc comparisons with Bonferroni corrections revealed that recognition of humans (*M* = .92, *SD* = .08) was significantly better than recognition of both paintings (*M* = .84, *SD* = .06), *p* = .002, and artificial races (*M* = .58, *SD* = .07), *p* < .001. Recognition of paintings was also significantly better than recognition of artificial races, *p* < .001. The effect of dimension was also significant regarding the categorization scores, *F*(2, 57) = 5.65, *p* = .006, $ƞ_{p}^{2}$ = .17, 90% CI = [.03, .29]. Post hoc comparisons using Bonferroni corrections indicating that participants categorized humans (*M* = .85, *SD* = .22) to a lesser extent than both paintings (*M* = .96, *SD* = .07), *p* = .031, and artificial races (*M* = .98, *SD* = .02), *p* = .009.


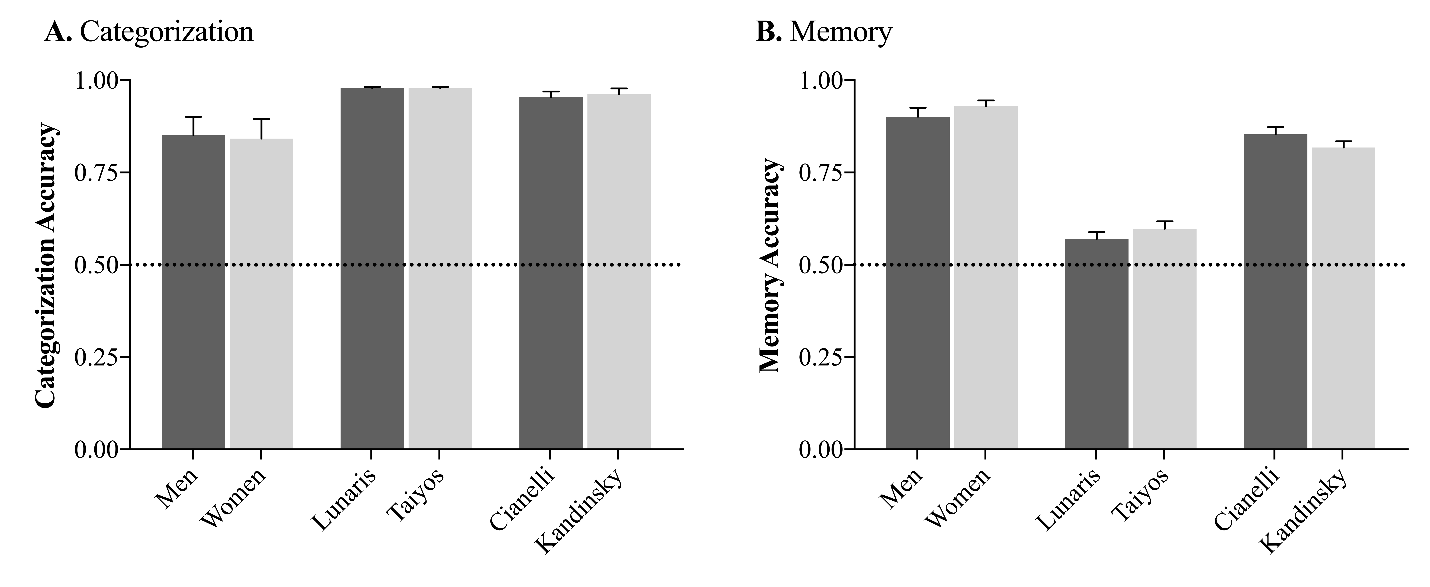


**Figure 1:** Accuracy scores in the categorization task and memory test for humans, artificial races and paintings.

References

Chua, K. W., Richler, J. J., & Gauthier, I. (2014). Becoming a Lunari or Taiyo expert: Learned attention to parts drives holistic processing of faces. *Journal of Experimental Psychology: Human Perception and Performance*, *40*(3), 1174–1182. https://doi.org/10.1037/a0035895

dos Santos Ferreira, P. R., da Cruz, S. A., Sampaio, W. M., Teodoro, J. V., Correia, L. L., & dos Santos, E. L. (2018). Interaction Between Equivalence and Categorization in the Recognition of Paintings. *The Psychological Record*, *68*(4), 477–488. https://doi.org/10.1007/s40732-018-0291-2

Lundqvist, D., Flykt, A., & Öhman, A. (1998). The Karolinska directed emotional faces (KDEF). *CD ROM from Department of Clinical Neuroscience, Psychology Section, Karolinska Institutet*, *91*, 630.

Schneider, W., Eschman, A., & Zuccolotto, A. (2002). *E-Prime User’s Guide*. Psychology Software Tools Inc.
